# Functional ability of community-dwelling older adults in Switzerland and its associations with multimorbidity and socioeconomic determinants: a longitudinal analysis of the Swiss Survey of Health, Ageing and Retirement in Europe (SHARE)

**DOI:** 10.21203/rs.3.rs-10693165/v1

**Published:** 2026-09-02

**Authors:** Beatriz Moreira, Jsabel Hodel, Yannick Rothacher, Carolina Fellinghauer, Renata Luethold, Roxanne Maritz, Clara Lussi, Carla Sabariego

**Affiliations:** University of Lucerne; Swiss Paraplegic Research; Swiss Paraplegic Research; Swiss Paraplegic Research; University of Bern; University of Lucerne; Swiss Paraplegic Research; University of Lucerne

**Keywords:** functional ability, disability, multimorbidity, healthy ageing, population health, older adults, health indicators, SHARE

## Abstract

Functional ability is central to healthy ageing, yet population-level evidence on functional ability in Switzerland remains limited as population ageing increases the demand for long-term care and rehabilitation services. Using longitudinal data from the Swiss Survey of Health, Ageing and Retirement in Europe (SHARE) and a validated functional ability score (0–100), we examined functional ability among community-dwelling adults aged 50 years and older. We investigated its association with multimorbidity, operationalized as both a binary disease count (defined as the presence of two or more chronic conditions) and a weighted disease burden score (0–10), over time using mixed-effects models. Two models were fitted accordingly and adjusted for age, sex, education, financial hardship, employment status, language, citizenship, living situation, and birth cohort. Our results showed that multimorbidity was consistently associated with lower functional ability across both operationalizations, with effects becoming more pronounced at older ages and among individuals experiencing financial hardship or with a lower level of education. This study provides a population-based, longitudinal description of functional ability among older adults in Switzerland, demonstrating that multimorbidity is negatively associated with functional ability in an age-dependent and socially patterned manner. These findings support functional ability as a policy-relevant indicator for monitoring healthy ageing and have direct implications for long-term care planning, rehabilitation, and equity-sensitive approaches to healthy ageing.

## Introduction

Population projections indicate that Switzerland’s population will continue to age rapidly. Around one in five people is currently aged 65 years or older, a proportion expected to rise to more than one in four by 2040, with increasing demand for health, social, long-term care (LTC), and rehabilitation services (1). Despite Switzerland’s high life expectancy, chronic health conditions are common in later life: almost half of community-dwelling adults aged 65 years or older report at least one chronic condition (2), and non-communicable diseases (NCDs) constitute the primary burden of disease (3). These demographic and epidemiological changes make it increasingly important to monitor health in later life in ways that go beyond survival and disease prevalence.

Reflecting this urgency, the 2021–2030 United Nations (UN) Decade of Healthy Ageing calls for coordinated action to address the challenges of population ageing. Healthy ageing has been defined as “*developing and maintaining the functional ability that enables well-being in older age*”(4). Functional ability is therefore central to the healthy ageing framework and is determined by the interaction between a person's intrinsic capacity — the physical and mental capacities they can draw on — and the environments in which they live, including the home, community, and broader social, built, attitudinal, and political contexts. While intrinsic capacity captures what an individual can draw on in terms of physical and mental reserves, functional ability goes further by reflecting how those capacities interact with the person's environment and can be translated into meaningful activities and participation in everyday life (4).

Evidence on the intrinsic capacity of ageing populations is extensive. Five subdomains – cognition, locomotion, vitality, psychology, and sensory capacity (vision and hearing) (5) – are considered pivotal for capturing the individual's intrinsic capacity at population level (6). These domains have been evaluated and validated longitudinally in different settings (7–9) and a recent systematic review covering 56 studies and more than 180’000 participants (10) showed the breadth of this evidence, while also highlighting substantial heterogeneity in how intrinsic capacity is operationalized across studies. Using data from the Survey of Health, Ageing and Retirement in Europe (SHARE), the intrinsic capacity construct has also been examined across 15 countries, and shown to be strongly associated with activities of daily living (ADL) and instrumental activities of daily living (IADL) losses, after adjustment for age, gender, education, income, and country (11).

In contrast, and despite its central role for healthy ageing, evidence on functional ability remains more limited. In 2025, a review attributed this gap to the lack of a globally standardised measurement tool to measure functional ability (12). Authors concluded that “*With only five years remaining in the Decade, finalising and implementing a standardised, person-centred measurement approach is urgent*.” Conceptually, however, functional ability is well established. In the WHO World Report on Ageing and Health (13), it is defined in broader terms, including five domains – meet basic needs; learn, grow and make decisions; be mobile; build and maintain relationships; and contribute to society. This is in close conceptual alignment with the WHO International Classification of Functioning, Disability and Health (ICF) (14), which understands functioning as the outcome of interactions between health conditions and the context of a person.

While a global standardised functional ability tool is not yet available, studies have operationalized functional ability in different ways. In Europe, longitudinal evidence has been generated from SHARE data, using ADL and IADL limitations to operationalize functional ability and document their predictors over time (15) as well as cohort-specific disability trajectories across European regions and birth cohorts (16). Other approaches have sought to construct broader functioning-based metrics that go beyond activities of daily living. The large Horizon 2020-funded ATHLOS project (17) adopted such an approach to operationalize functional ability and identify healthy ageing trajectory patterns and their determinants (18). ATHLOS harmonized data from 16 ageing cohorts, creating a pooled dataset of 343'915 individuals, implemented the ICF as a reference framework and used Item Response Theory (IRT) to generate a healthy ageing metric using 41 items, including body functions as well as ADLs and IADLs (17). Similarly drawing on the ICF framework for item selection, a SHARE-specific functional ability metric was developed using a Rasch-based approach (19). This metric was subsequently applied to model disability severity transitions and their predictors in the Swiss ageing population (20).

The availability of such metrics creates new opportunities to examine how key health-related factors shape functional ability over time. Multimorbidity, commonly defined as the coexistence of multiple chronic diseases (21), is an important public health concern in the context of population ageing as it is associated with greater clinical complexity and care needs, making it highly relevant for health-system planning (22). In Switzerland, prevalence estimates differ significantly depending on definitions, the population studied, and the data sources used (25); among community-dwelling individuals, multimorbidity affects 25% of those aged 65–79 years old, rising to 41% among those aged 80 years or older (2). Moreover, multimorbidity has been shown to be a key predictor of functional ability across populations and settings, and is associated with less favourable healthy ageing trajectories and greater functional decline (18, 23, 24).

Given the role of functional ability in healthy ageing, studies using available data to describe it over time are essential. Functional ability data are available in Swiss surveys (26) as well as the previously mentioned functional ability metric built with data from SHARE (19). However, to the best of our knowledge, no study has yet longitudinally modelled the functional ability of community-dwelling older adults in Switzerland and examined its association with multimorbidity. Addressing this gap is critical for understanding dynamic health needs and informing LTC and rehabilitation planning. The objective of this study is therefore to (i) describe functional ability among adults aged 50 years or older living in the community in Switzerland and (ii) examine the association of functional ability with multimorbidity over time, using longitudinal data from the Swiss SHARE. By focusing on functional ability as the core outcome of healthy ageing, our study aims to support evidence-informed and ageing-sensitive policy making and service planning.

## Methods

### Study design and population

This study used data from the Swiss sample of the SHARE (Release 9.0.0, accessed October 2024) (27–34). SHARE is a multidisciplinary, cross-national panel survey carried out every two years since 2004, collecting information on health, socio-economic circumstances, and social networks. The target population includes individuals aged 50 years or older and their spouse/partner irrespective of age. In Switzerland, interviews are conducted in German, French, and Italian. Individuals are ineligible if incarcerated, hospitalized, abroad for the entire fieldwork period, unable to complete the interview in an eligible language, or moved to an unknown address. Switzerland currently contributes nine waves of data collection (2004–2022); details on design and fieldwork are described elsewhere (35, 36). For this study, we used data from waves 1–9, excluding wave 3 (SHARELIFE), as it lacks the required variables. Additional study-specific exclusions were applied: (i) observations without a valid interview (neither main interview nor end-of-life proxy interview), (ii) participants younger than 50 years at study entry, and (iii) participants without a valid functional ability score in any wave.

### Measures

#### Primary Outcome

Functional ability was measured using an existing SHARE-based metric previously developed and validated (19). The metric was constructed by first linking SHARE items to the ICF to identify relevant functional ability content - an approach also used in other large ageing cohort studies, such as ATHLOS (17) - and then applying modern test theory methods, specifically Rasch analysis using the Partial Credit Model (37), to derive an interval-scaled total score. The Rasch analysis evaluated item fit, local item dependency, and unidimensionality, supporting the selection of items that could be treated as indicators of a single underlying construct of functional ability, with the resulting score demonstrating good reliability and measurement properties.

The metric includes 33 SHARE items covering both body functions and activities and participation domains. Body-function items include, for example, sleeping problems, hearing, energy and drive, and pain, while activities and participation items cover domains such as mobility, self-care, domestic life, and IADLs. The resulting score ranges from 0 to 100, with higher values indicating better functional ability. As the metric is interval-scaled, it allows differences in functional ability to be compared at both the individual and population level.

According to the Rasch analysis, in a population with declining functional ability scores, we would expect that, on average, people tend to have problems or limitations in items in order of their increasing difficulty. Scores above 70 points reflect, on a population level, relatively preserved functional ability, with most difficulty concentrated in vision and hearing - domains that remain commonly affected even among otherwise high-functioning individuals. Scores around 50–65 tend to show, in addition to limitations in vision and hearing, increasing difficulties in basic ADLs, concentration, upper body strength, fatigue, and mobility. Lower scores, around or below 40 points, indicate, in addition to the difficulties described above, increasing difficulty with more basic physical and IADL (e.g., eating, hand use, sitting, transferring). Further details on item selection, model fit, reliability, and score interpretation are available in the original validation study (19).

### Main explanatory variables

#### Multimorbidity

Multimorbidity was defined using the following SHARE-reported conditions (ever diagnosed/currently having): heart attack (including myocardial infarction and coronary thrombosis) or other heart problems (including congestive heart failure), stroke/cerebrovascular disease, diabetes/high blood sugar, chronic lung disease, asthma, cancer or malignant tumor (excluding minor skin cancers), stomach/duodenal/peptic ulcer, Parkinson’s disease, cataracts, hip fracture, other fractures, Alzheimer’s disease/dementia/organic brain syndrome/senility or other serious memory impairment, affective/emotional disorders, arthritis, rheumatoid arthritis, osteoarthritis, and chronic kidney disease. Clinical depression was defined as a score ≥ 4 on the European Depression Scale (EURO-D) (38).

Acknowledging that simple disease counts do not account for differences in disease severity or burden, multimorbidity was operationalized in two ways: first, as a binary count variable indicating the presence of two or more conditions, and second, as a continuous disease burden score informed by Global Burden of Disease (GBD) disability weights (39). Following a previously published approach (20), each SHARE condition was mapped to corresponding GBD health states and severity levels. Disability weights, which represent the amount of health loss associated with a condition on a scale from 0 to 1, were averaged across severity levels to obtain one weight per SHARE condition. For individuals with more than one condition, these weights were combined using the standard GBD multiplicative formula for cumulative disability weights, ensuring that combined burden increased with each additional condition while remaining on the 0–1 scale. The resulting combined disability weight was multiplied by 10 to obtain a continuous multimorbidity burden score ranging from 0 to 10, with higher values indicating greater disease burden. Further details on the mapping of SHARE conditions to GBD health states and disability weights are provided elsewhere (20). Hypertension, high cholesterol, and osteoporosis were not included in the burden score or analyses because they are considered risk factors rather than health states in the GBD framework, but they are reported descriptively.

#### Covariates

Covariates were selected based on their established associations with functional ability, multimorbidity, or both in older adult populations. Age at interview (in years) and birth cohort (≤ 1939; 1940–49; 1950–59; 1960–69; ≥1970) were included to account for within-person ageing effects and between-cohort differences in baseline health, respectively. Sex (male/female) was included given well-documented sex differences in functional limitations and multimorbidity (40). Interview language (German/French/Italian) was used as a pragmatic proxy for region of residence, given known regional differences in health outcomes across Switzerland's language regions. Education level (low/medium/high, ISCED 1997) and financial hardship (from the question: “*thinking of your household's total monthly income, would you say that your household is able to make ends meet?*”, yes/no) were included as indicators of socioeconomic position, which is a well-established determinant of multimorbidity and may influence how chronic conditions and treatment burden translate into functional limitations (41). Additionally, they can serve as indicators of the ability of older persons to meet basic needs, one of the key domains of functional ability (42). Living situation (living alone for household size < 2 vs. not living alone for household size ≥ 2) was included as a proxy for social support, and Swiss citizenship (yes/no) to account for migrant health differences. Employment status (employed or self-employed; homemaker; retired; unemployed; permanently sick or disabled; other) was treated as a separate covariate rather than as an indicator of socioeconomic position, consistent with evidence that employment status may not represent occupational disadvantage itself (43).

#### Missing data management and imputation

All non-substantive response categories (e.g., “don’t know”, “refusal”, etc) were recoded as missing. Prior to applying the study-specific exclusion criteria, we manually imputed time constant sociodemographic variables (education level, language, and citizenship) that are assessed at baseline but that appear longitudinally in later waves with missing values. Missing values for age at interview were reconstructed whenever possible using other SHARE information (e.g., date of birth and interview date).

Because the set of self-reported health conditions assessed in SHARE varies across waves, not all conditions are observed at every measurement occasion. To improve cross-wave comparability of multimorbidity measures, we applied a multi-step strategy. First, conditions were classified as chronic (e.g., diabetes) versus temporary (e.g., hip fracture) (see full description in Table S1 in the Supplemental File). For chronic conditions, we imposed longitudinal consistency by carrying a first reported occurrence forward across subsequent waves and carrying the last confirmed non-occurrence backwards across earlier waves. After this step, missing values remained only in the health conditions and financial hardship variables; these were then single-imputed using MissForest, a non-parametric random-forest-based imputation method (44). The multimorbidity variables were then constructed from the respective imputed datasets.

An overview of variables included in the single imputation procedure (Table S1), of missing observations across model variables and health conditions (Figures S1–S2), and a correlation matrix of model variables (Figure S3) can be found in the Supplemental File.

### Statistical analysis

Descriptive statistics were summarized at baseline, defined as each participant’s first available observation. We first conducted an initial data analysis, guided by the approach described by Lusa et al. (45). To estimate longitudinal associations between functional ability and multimorbidity, we fitted linear mixed-effects models to account for repeated measurements within individuals. Two models were fitted, each with identical covariate specifications but different operationalizations of the main explanatory variable: Model A included the continuous operationalization of multimorbidity (burden) and Model B included the binary operationalization of multimorbidity (count).

The models included a participant-specific random intercept and a set of fixed effects (see Supplemental File for full model formulas). Covariates (including decisions on main effects, interactions, and non-linear terms) were selected a priori based on evidence and expert input and discussed within the project team. We hypothesized that the association of multimorbidity with functional ability may vary with age; therefore, age was modelled using both linear and quadratic terms to capture potential non-linear relationships. To test whether the association between multimorbidity and functional ability differed across population subgroups, we included interactions between the main variable of interest and sex, education, financial hardship, and interview language, as well as an interaction between the main variable of interest and age. Remaining covariate variables (citizenship, living situation, employment status, and birth cohort) were entered as main effects. Model assumptions including normally distributed residuals and normally distributed random intercepts were checked using graphical visualizations (residual plots and Q-Q plots). To aid interpretation of the fitted models and interaction terms, results were visualized using effect plots showing predicted functional ability over time for representative combinations of covariate values. Analyses were conducted in R using the *nlme* package (46). The reporting of this study followed the guidelines about Strengthening the Reporting of Observational Studies in Epidemiology (STROBE) (47).

## Results

### Sample characteristics

In total, 25’863 observations from 7’178 Swiss participants were obtained from SHARE. After excluding participants without a valid interview (N = 2’250), aged < 50 years at baseline (N = 480), or without a functional ability score (N = 8), the final sample included 4’440 individuals and 16’962 observations. Descriptive information of the study sample is presented at baseline and for different disease burden values (Table 1; see supplemental Tables S2-S6 for other covariate groups). The median age was 62 years (IQR = 55–71). Most participants were interviewed in German (71%), had Swiss citizenship (92%), and a medium education level (60%). At baseline, 45% were employed or self-employed, 41% retired, 17% reported financial hardship, and 22% lived alone. Regarding health status, 30% met the binary multimorbidity criterion (≥ 2 conditions), while the disease burden score had a median of 0.5 (IQR = 0.0–3.0) and a mean of 1.6 (SD = 1.9). Functional ability scores declined across burden groups, from a median of 72.1 (IQR = 68.4–82.1) in the no burden group to 60.4 (IQR = 54.9–65.3) in the high burden group.

Using the GBD weighted multimorbidity score, participants were grouped into four multimorbidity burden categories based on the empirical distribution of the score (range 0–10): no burden (score = 0), low burden (> 0–1.6), medium burden (> 1.6–3.4), and high burden (> 3.4), corresponding to the median and 75th percentile of the observed score distribution (maximum observed score = 9). These thresholds are illustrative of the score distribution and do not represent validated clinical cut-points. Figure 1 shows the prevalence of health conditions at baseline across the four burden groups: in the lowest burden group, almost all participants had only one health condition, with cataracts predominating; as burden increased, participants accumulated two to eight or more conditions, with arthritis and depression becoming increasingly prevalent in the moderate and high groups with depression exceeding 60% in the highest burden group. Over half of participants in the moderate burden group still had only one condition, illustrating how the composition of multimorbidity shifts substantially with increasing burden even when condition counts remain as no multimorbidity. Overall, arthritis and depression were the most prevalent conditions when stratified by multimorbidity count. Further descriptive results are provided in the Supplemental File (Tables S7–S8; Figures S4–S9).

### Mixed effects modelling

Table 2 shows the results of the linear mixed-effects models examining the longitudinal associations between functional ability and the different operationalizations of multimorbidity. Model assumption checks can be found in the Supplemental File.

The models included interaction terms, indicating that the association between multimorbidity and functional ability varies depending on other covariates and therefore cannot be summarized by a single coefficient. Overall, multimorbidity was negatively associated with functional ability across models A and B. The pattern of significant interactions was similar for both models: multimorbidity burden and count both showed significant interactions with age (burden×age and burden×age^2^: p < 0.001; count×age and count×age^2^: p < 0.001), indicating that the negative association with functional ability becomes more pronounced with advancing age in a non-linear way. Financial hardship also significantly modified this association (burden×financial hardship: p < 0.001; count×financial hardship: p < 0.001), with a stronger negative effect of multimorbidity on functional ability among individuals reporting financial difficulty. For education level, evidence for effect modification was driven mainly by a negative association for low versus high education (Model A interaction: p = 0.045; Model B interaction: p = 0.039). There was no evidence that the multimorbidity-functional ability association differed by sex or language region.

### Effect plots

Effect plots summarize the association between a selected variable and the outcome while holding all other covariates constant at specific values. Figure 2 shows the average predicted functional ability scores across age by multimorbidity (burden and count) for two reference profiles born in 1950–59 (see Supplemental Figures S14 and S15 for other cohorts) with contrasting covariate value combinations:

#### Scenario 1

male, high education level, without financial difficulty, German-speaking, Swiss citizenship, not living alone, employed or self-employed;

#### Scenario 2

female, low education level, with financial difficulty, Italian-speaking, no Swiss citizenship, living alone, permanently sick or disabled.

Across panels, predicted functional ability scores follow a non-linear age pattern, peaking around age 60–70 before declining more steeply at older ages. Higher multimorbidity (burden or count) is consistently associated with lower predicted functional ability at all ages. Moreover, scenario 2 profiles, which have more socially disadvantaged covariates combinations, lie below scenario 1 even among those with no multimorbidity. For example, in panel A, among those with no multimorbidity (blue line), at age 65, predicted functional ability was on average 12 points lower in scenario 2 than in scenario 1 (64.9 [95% CI: 63.0–66.8] vs 78.1 [95% CI: 77.3–78.9]) and 13 points lower at age 80 (61.2 [95% CI: 59.2–63.2] vs 74.4 [95% CI: 73.4–75.4]). The gap was largest for high multimorbidity burden (red line), where scenario 2 was on average 19 points lower than scenario 1 at both age 65 (43.5 [95% CI: 40.7–46.4] vs 62.3 [95% CI: 60.6–64.0]) and age 80 (40.4 [95% CI: 37.5–43.4] vs 59.2 [95% CI: 57.4–61.0]). Comparing scenario 1 without multimorbidity to scenario 2 with high multimorbidity burden, the difference was 34 points for both ages. Similar patterns were observed for multimorbidity count (Panel B). See Supplemental Figure S16 for predicted functional ability across the full range of multimorbidity scores by age groups.

## Discussion

This study described functional ability among adults aged 50 years or older living in the community in Switzerland and examined the association of functional ability, a key healthy ageing construct, with multimorbidity over time. We found a strong negative association between two operationalizations of multimorbidity, namely disease count and disease burden, and functional ability, with age-related declines following a non-linear pattern and becoming steeper at older ages. Importantly, the multimorbidity-functional ability association was socially patterned: financial hardship and low education were associated with lower functional ability and amplified the negative association between multimorbidity and functional ability. Beyond these findings, the study demonstrates the feasibility and value of using existing Swiss survey data to examine functional ability longitudinally at the population level, complementing evidence on intrinsic capacity. Moreover, the operationalization of functional ability using the ICF as a reference framework is in line with the recently launched WHO Framework for implementing a life course approach to health (48). Our study shows therefore that the lack of a globally standardised tool to measure functional ability is a barrier that can be overcome using available population data and modern test theory, generating the evidence needed to timely monitor the implementation of the UN Decade of Healthy Ageing.

Our findings highlight the importance of addressing multimorbidity and chronic disease burden in ageing societies. In our models, functional ability appeared relatively stable or rising slightly between ages 50 and 65 before declining after approximately 70. However, this counterintuitive pattern of an apparent increase in functional ability before the age of 65 should be interpreted with caution and might not reflect true population-level improvement with age. For the highest burden group, it likely reflects sparse data and survival bias - individuals with the highest burden are less likely to survive to older ages. Cohort composition also contributes: when examined separately by birth cohort and restricted to each cohort’s own observed age range, individual cohorts showed mostly flat or declining trajectories (see Figure S17 in the Supplemental File), supporting this interpretation. The decline after approximately 70 is therefore the more robust finding, consistent with Switzerland's high life expectancy and overall healthy older population (49, 50). This suggests that functional losses are concentrated at older ages, when accumulated morbidity and age-related decline compound each other. Because our sample included relatively few participants aged 80 years or older (less than 10%), the association between multimorbidity and functional ability may be even more pronounced in the oldest-old (51). In this context, Switzerland’s policy focus on NCD prevention is highly relevant (52) but equivalent emphasis should be placed on disease management of NCDs, including multimorbidity, for instance through timely rehabilitation aimed at optimizing functional ability and reducing LTC needs (53).

Despite Switzerland's favourable population health indicators and high standard of living, our findings indicate a social gradient in functional ability that mirrors patterns well documented in the broader social determinants of health literature. Financial hardship, lower education, being unemployed, being permanently sick or disabled, and living alone were associated with lower predicted functional ability. Moreover, financial hardship and lower education amplified the negative association between multimorbidity and functional ability, aligning with evidence that social disadvantage shapes not only the risk of developing multimorbidity (54) but also the extent to which it translates into limitations in daily life (55, 56). This is important as Switzerland’s high out-of-pocket healthcare costs may exacerbate inequities in access to care (57), particularly among individuals experiencing financial hardship. In addition, the predicted trajectories indicate that disadvantaged groups start from lower levels of functional ability and remain lower over time, consistent with findings from healthy-ageing trajectory research (58). The sensitivity of the functional ability score, operationalized using the conceptual framework of the ICF, to well-established social gradients (59) also supports its ability to describe meaningful inequalities even within a high-income context. Overall, our findings suggest that social disadvantage and multimorbidity are jointly associated with lower functional ability, underscoring the need for stratified and equity-sensitive approaches to healthy ageing. However, these associations should not be interpreted causally, as the relationship between socioeconomic factors and functional ability is likely bidirectional.

The disease burden score offers a more nuanced measure that is sensitive to both the number and the relative severity of conditions. This aligns with prior evidence that weighted multimorbidity indexes perform better than counts when the goal is to summarize overall burden relevant to patient-centred outcomes and policy-making (60). The disease burden score distinguishes groups with different health profiles, whereas the binary count operationalization tends to collapse heterogeneous individuals (high vs low burden) into a single “multimorbidity” category. The latter is less precise than the former and can dilute differences - especially for those with the most severe disease burden – leading to an overlook of this population, who is at high risk of severe disability. Indeed, a recent study describing disability severity transitions in the ageing population in Switzerland reported that the operationalization as burden might have the potential to provide decision-makers with more nuanced information than just focusing on the count of diseases (20). As the high multimorbidity burden group is probably the group with the highest need for expensive LTC services, this nuance matters in light of planning services in financially challenged health and social systems (52).

A key contribution of this study is to demonstrate that functional ability can be modelled longitudinally using existing Swiss survey data, providing a population-level indicator that complements morbidity and mortality monitoring. This longitudinal perspective is central to a life course approach to healthy ageing, which emphasizes that functional ability trajectories are shaped by cumulative exposures and by critical and sensitive transitions across life stages, with implications for when and where prevention and support can most effectively be targeted (53). From a policy perspective, the functional ability indicator may help identify groups at higher risk of decline and support the targeting of preventive interventions or the allocation of rehabilitation resources among older adults, as well as the estimation and forecasting of LTC needs and overall health planning. More broadly, routine monitoring of functional ability can shift policy discussions from curative strategies or survival alone toward outcomes that matter to people in daily life. Because functional ability is shaped not only by health conditions but also by wider social contexts, these data can also help inform public health measures beyond the health system, consistent with calls for stronger links between health policy and domains such as education, employment, social welfare, and the environment (61).

## Limitations

This study has also limitations. First, direct comparisons between multimorbidity burden and multimorbidity count are inherently constrained because they differ in scale (continuous vs binary) and therefore capture different types of variation. We also did not explore alternative multimorbidity conceptualizations (e.g., disease clusters or data-driven groups), but the consistency of findings across both measures supports the robustness of our conclusions. Second, our imputation strategy assumes data are missing at random (MAR). Given that missingness was largely attributable to survey design features, and we used logically consistent, rule-based replacements where feasible, the MAR assumption is plausible, but bias cannot be fully excluded. Third, attrition and survivor bias are inherent challenges in longitudinal studies of older adults: individuals with worse functional ability are more likely to drop out or die over follow-up, potentially leading to an underestimation of the true extent of functional decline in the most vulnerable groups. Fourth, our sample overrepresents Swiss citizens (≈ 90% in Swiss SHARE vs. 72–73% in national statistics (62), limiting representativity since migrants may face different health and socio-economic risks. Fifth, while SHARE includes spouses/partners living in the same household, we did not account for potential clustering at the household level. Finally, generalisability beyond the Swiss context may be limited, and the multimorbidity burden score relies on a specific disease-weighting scheme that may not translate across settings.

## Conclusion

Our study shows that the lack of a globally standardised tool to measure functional ability is a barrier that can be overcome using available population data and modern test theory to longitudinally model functional ability, generating the evidence needed to timely monitor the implementation of the UN Decade of Healthy Ageing. The monitoring of functional ability, alongside morbidity, can inform policy by improving risk stratification and supporting more targeted prevention, rehabilitation, and resource allocation. Further work incorporating contextual and environmental determinants will be important to strengthen policy relevance.

## Supplementary Material

This is a list of supplementary files associated with this preprint. Click to download.

- SupplementalFileFinalblinded.docx

## Figures and Tables

**Figure 1:**
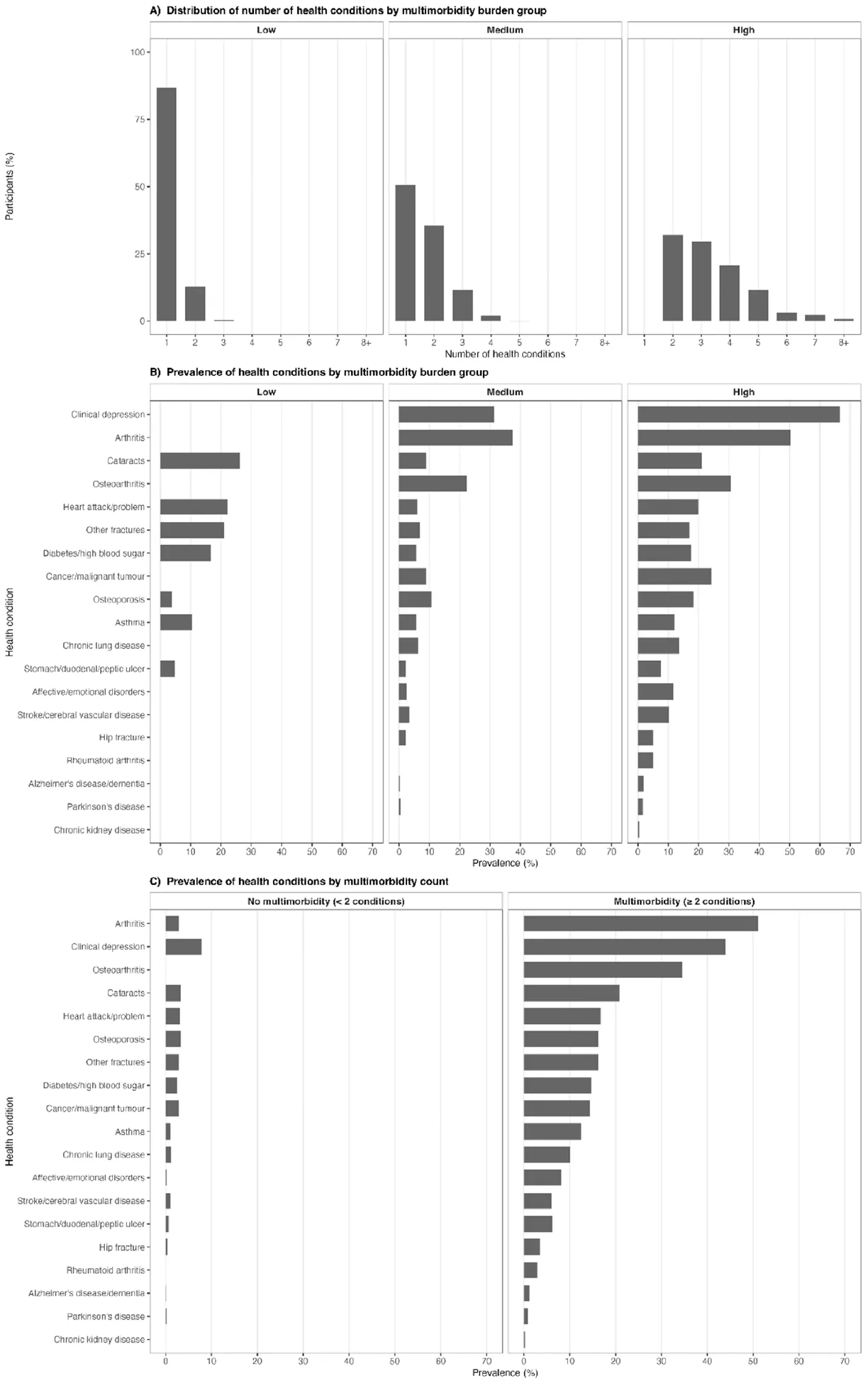
Overview of the distribution and prevalence of health conditions, at baseline. **(A)** Distribution of the number of reported health conditions across multimorbidity burden groups defined by the weighted multimorbidity score (0–10): low (0–1.6], medium (1.6–3.4], and high (>3.4). **(B)** Prevalence (%) of the most common health conditions within each multimorbidity burden group (low/medium/high). **(C)** Prevalence (%) of the most common health conditions by multimorbidity count (No vs Yes). Burden groups were defined using representative values from the empirical score distribution (0–10 scale): no burden (score = 0, minimum), low burden (0–1.6], medium burden (1.6–3.4], and high burden (>3.4). The upper boundaries of the low and medium groups correspond to the median (1.6) and the 75^th^ percentile (3.4) of the score distribution, respectively, and the maximum observed score was 9. These values are illustrative of the distribution and do not represent analytical cut-off points.

**Figure 2:**
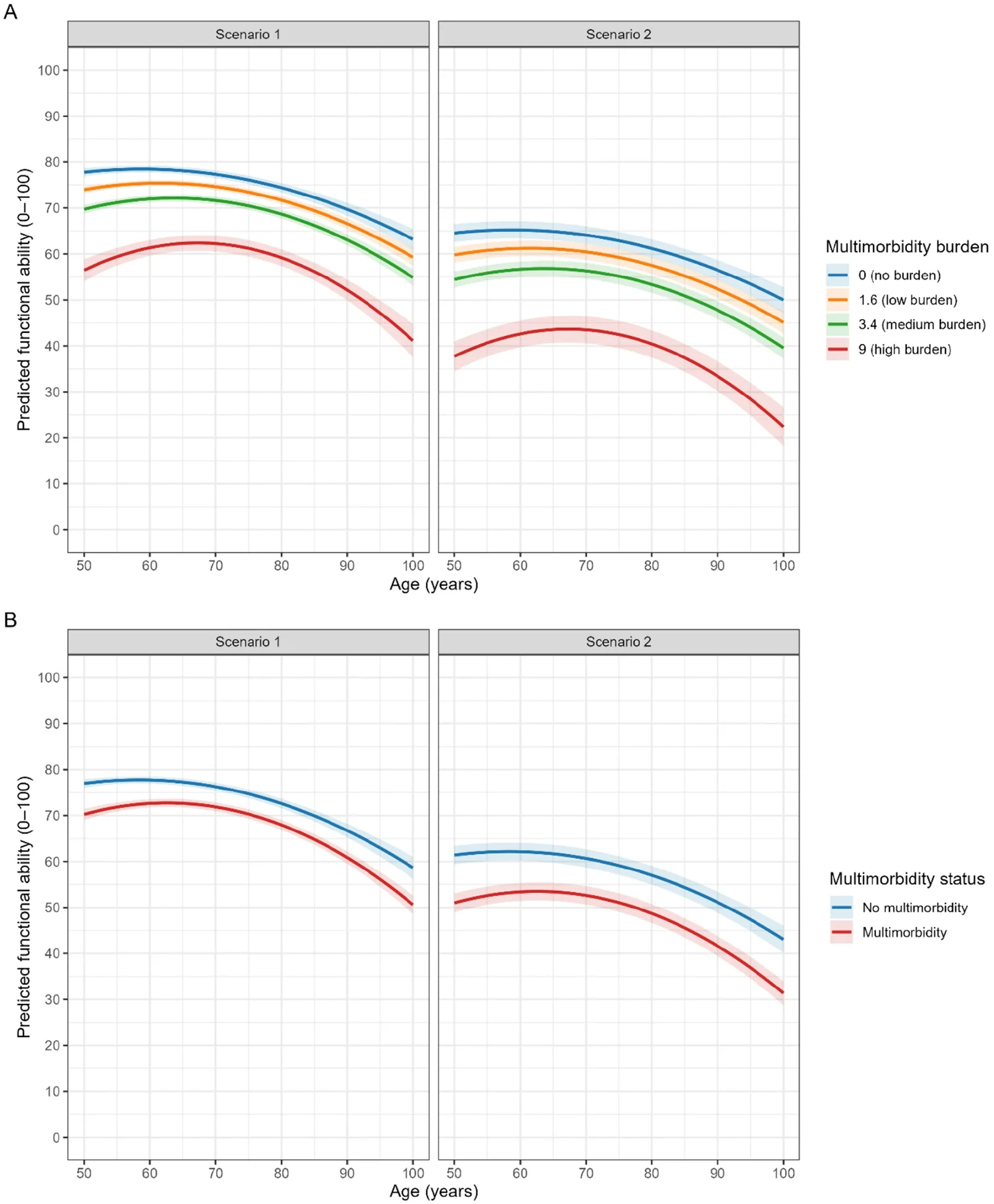
**(A)** Predicted functional ability scores (0–100 scale) across ages 50–100 by multimorbidity burden (score ranging 0–10, with higher values indicating greater burden). Four representative score values were selected based on the empirical distribution of the sample: 0 (no burden, minimum), 1.6 (low burden, median), 3.4 (moderate burden, 75^th^ percentile), and 9.0 (high burden, maximum observed score). These values are illustrative of the score distribution and do not represent analytical cut-off points. **(B)** Predicted functional ability scores (0–100 scale) across ages 50–100 by multimorbidity count (binary: no multimorbidity vs. multimorbidity). Panels show predictions for contrasting covariate combinations: scenario 1 (male, high education, no financial difficulty, German-speaking, Swiss citizenship, not living alone, employed or self-employed, born in 1950–59) and scenario 2 (female, low education, financial difficulty, Italian-speaking, no Swiss citizenship, living alone, permanently sick or disabled, born in 1950–59). Lines represent predicted values; shaded areas represent 95% confidence intervals of the mean (expected) functional ability outcome. These intervals do not include residual or random-effect variability and therefore should not be interpreted as prediction intervals for individual observations.

**Table 1:** Overview characteristics of participants at baseline interview, overall and stratified by different multimorbidity burden groups.

Characteristics	Baseline (N = 4440)	No burden group (N = 2179)	Low burden group (N = 483)	Medium burden group (N = 1004)	High burden group (N = 774)	Missing (%)
SHARE wave, n (%)						0.0
1	938 (21.1)	495 (22.7)	106 (22.0)	211 (21.0)	126 (16.3)	
2	698 (15.7)	383 (17.6)	71 (14.7)	139 (13.8)	105 (13.6)	
4	2502 (56.4)	1145 (52.6)	288 (59.6)	578 (57.6)	491 (63.4)	
5	13 (0.3)	5 (0.23)	3 (0.6)	3 (0.3)	2 (0.3)	
6	7 (0.2)	6 (0.3)	0 (0.0)	1 (0.1)	0 (0.0)	
7	7 (0.2)	3 (0.1)	0 (0.0)	1 (0.1)	3 (0.4)	
8	188 (4.2)	96 (4.4)	10 (2.1)	52 (5.2)	30 (3.9)	
9	87 (2.0)	46 (2.1)	5 (1.0)	19 (1.9)	17 (2.2)	
Age at interview*, median [IQR]	62 [55, 71]	59.00 [54; 67]	66 [58; 74]	63 [56; 72]	66 [57; 75]	0.0
Age at interview*, n (%)						0.0
50–59	1892 (42.6)	1105 (50.7)	144 (29.8)	405 (40.3)	238 (30.8)	
60–69	1319 (29.7)	639 (29.3)	155 (32.1)	294 (29.3)	231 (29.8)	
70–79	853 (19.2)	336 (15.4)	126 (26.1)	202 (20.1)	189 (24.4)	
80+	376 (8.5)	99 (4.5)	58 (12.0)	103 (10.3)	116 (15.0)	
Sex=Female, n (%)	2348 (52.9)	1086 (49.8)	193 (40.0)	598 (59.6)	471 (60.9)	0.0
Education level*, n (%)						1.0
Low	1040 (23.4)	454 (20.8)	101 (20.9)	252 (25.1)	233 (30.1)	
Medium	2644 (59.6)	1352 (62.1)	278 (57.6)	584 (58.2)	430 (55.6)	
High	756 (17.0)	373 (17.1)	104 (21.5)	168 (16.7)	111 (14.3)	
Interview language*=FR/IT, n (%)	1279 (28.8)	536 (24.6)	117 (24.2)	324 (32.3)	302 (39.0)	0.0
Employment status, n (%)						0.5
Employed or self-employed	2005 (45.2)	1209 (55.5)	173 (35.8)	415 (41.3)	208 (26.9)	
Homemaker	362 (8.2)	170 (7.8)	35 (7.3)	102 (10.2)	55 (7.1)	
Retired	1830 (41.2)	740 (34.0)	255 (52.8)	424 (42.2)	411 (53.1)	
Permanently sick or disabled	118 (2.7)	16 (0.7)	6 (1.2)	30 (3.0)	66 (8.5)	
Unemployed	87 (2.0)	30 (1.4)	9 (1.9)	22 (2.2)	26 (3.4)	
Other	38 (0.9)	14 (0.6)	5 (1.0)	11 (1.1)	8 (1.0)	
Citizenship*=Yes, n (%)	4075 (91.8)	2000 (91.8)	443 (91.7)	922 (91.8)	710 (91.7)	0.2
Financial hardship*=Yes, n (%)	735 (16.6)	258 (11.8)	65 (13.5)	185 (18.4)	227 (29.3)	28.6
Living alone = Yes, n (%)	990 (22.3)	431 (19.8)	101 (20.9)	232 (23.1)	226 (29.2)	0.0
High blood pressure = Yes, n (%)	1250 (28.2)	480 (22.0)	158 (32.7)	307 (30.6)	305 (39.4)	0.2
High blood cholesterol = Yes, n (%)	640 (14.4)	208 (9.6)	94 (19.5)	155 (15.4)	183 (23.6)	0.2
Osteoporosis = Yes, n (%)	321 (7.2)	52 (2.4)	19 (3.9)	108 (10.8)	142 (18.4)	63.2
Multimorbidity (count)*=Yes, n (%)	1334 (30.1)	0 (0.0)	64 (13.3)	496 (49.4)	774 (100.0%)	0.0
Multimorbidity (burden)*, median [IQR]	0.5 [0.0; 3.0]	0.0 [0.0; 0.0]	1.0 [0.7; 1.4]	3.0 [2.2; 3.0]	4.6 [4.1; 5.4]	0.0
Multimorbidity (burden)*, mean (SD)	1.6 (1.9)	0.0 (0.0)	1.1 (0.4)	2.6 (0.5)	4.9 (1.0)	0.0
Functional ability score, median [IQR]	72.1 [62.7; 76.5]	72.1 [68.4, 82.1]	72.1 [65.3, 76.5]	68.4 [60.4, 72.1]	60.4 [54.9, 65.3]	0.0

Note: Burden groups were defined using representative values from the empirical score distribution (0–10 scale): no burden (score = 0, minimum), low burden (0–1.6], medium burden (1.6–3.4], and high burden (> 3.4). The upper boundaries of the low and medium groups correspond to the median (1.6) and the 75th percentile (3.4) of the score distribution, respectively, and the maximum observed score was 9. These values are illustrative of the distribution and do not represent analytical cut-off points. Baseline refers to each person's first SHARE interview. IQR, interquartile range; SD, standard deviation; FR, French; IT, Italian; SHARE; Survey of Health, Ageing, and Retirement in Europe.

* After missing data management. Missing values reported here reflect the pre-single imputation dataset.

**Table 2:** Fixed and random effects estimates from linear mixed-effects models predicting functional ability scores (0–100) by multimorbidity burden (Model A) and multimorbidity count (Model B), with interaction terms (denoted by x) with selected covariates. Results are reported as coefficients with standard errors (SE), 95% confidence intervals (CI), and p-values. Statistically significant associations (p < 0.05) are shown in bold.

Variables	Model A – Multimorbidity burden			Model B – Multimorbidity count		
	Coefficient (SE)	95% CI	p-value	Coefficient (SE)	95% CI	p-value
**Fixed effects**						
Intercept	**43.32 (4.60)**	[34.30, 52.34]	< 0.001	**36.12 (4.70)**	[26.91, 45.32]	< 0.001
Multimorbidity (burden)	−**8.617 (1.357)**	[−11.277, −5.957]	< 0.001			
Multimorbidity (count): Yes (ref: No)				−**31.09 (6.12)**	[−43.08, −19.10]	< 0.001
Age	**1.05 (0.13)**	[0.79, 1.32]	< 0.001	**1.29 (0.14)**	[1.02, 1.56]	< 0.001
Age^2^	−**0.01 (0.00)**	[−0.01, −0.01]	< 0.001	−**0.01 (0.00)**	[−0.01, −0.01]	< 0.001
Sex: Female (ref: male)	−**1.29 (0.26)**	[−1.80, −0.78]	< 0.001	−**1.57 (0.27)**	[−2.09, −1.04]	< 0.001
Education level (ref: High)						
Low	−**2.53 (0.43)**	[−3.36, −1.69]	< 0.001	−**2.81 (0.44)**	[−3.66, −1.95]	< 0.001
Medium	−**1.06 (0.35)**	[−1.74, −0.38]	0.002	−**1.30 (0.36)**	[−1.99, −0.60]	< 0.001
Financial hardship: No (ref: Yes)	0.45 (0.32)	[−0.18, 1.07]	0.159	0.62 (0.32)	[−0.01, 1.24]	0.053
Language (ref: German)						
French	**0.62 (0.31)**	[0.02, 1.23]	0.043	0.07 (0.32)	[−0.55, 0.69]	0.833
Italian	−1.24 (0.65)	[−2.51, 0.03]	0.055	−1.10 (0.67)	[−2.42, 0.22]	0.102
Citizenship: Yes (ref: No)	0.78 (0.40)	[−0.00, 1.56]	0.051	**0.88 (0.43)**	[0.05, 1.72]	0.039
Living Situation: Not living alone (ref: Living alone)	**0.40 (0.21)**	[−0.00, 0.81]	0.052	**0.54 (0.22)**	[0.11, 0.97]	0.014
Employment status (ref: Employed/Self-employed)						
Homemaker	0.29 (0.30)	[−0.29, 0.88]	0.328	0.16 (0.31)	[−0.45, 0.77]	0.604
Permanently sick or disabled	−**6.52 (0.53)**	[−7.56, −5.47]	< 0.001	−**8.08 (0.55)**	[−9.16, −7.01]	< 0.001
Retired	0.15 (0.22)	[−0.28, 0.58]	0.490	−0.01 (0.23)	[−0.46, 0.44]	0.958
Unemployed	−**2.03 (0.62)**	[−3.25, −0.80]	0.001	−**2.71 (0.65)**	[−3.98, −1.44]	< 0.001
Other	−1.19 (0.66)	[−2.49, 0.11]	0.072	−1.28 (0.68)	[−2.62, 0.06]	0.060
Birth cohort (reference: ≤1939)						
1940–49	**1.71 (0.32)**	[1.08, 2.34]	< 0.001	**1.29 (0.34)**	[0.61, 1.96]	< 0.001
1950–59	**2.55 (0.38)**	[1.80, 3.29]	< 0.001	**2.04 (0.40)**	[1.25, 2.83]	< 0.001
1960–69	**2.59 (0.53)**	[1.55, 3.63]	< 0.001	**1.95 (0.57)**	[0.84, 3.06]	< 0.001
≥1970	**14.37 (6.38)**	[1.87, 26.87]	0.024	**13.70 (6.75)**	[0.47, 26.93]	0.042
Multimorbidity (burden) × Age	**0.18 (0.04)**	[0.10, 0.26]	< 0.001			
Multimorbidity (burden) × Age^2^	−**0.001 (0.00)**	[−0.002, −0.001]	< 0.001			
Multimorbidity (burden) × Sex (female)	0.03 (0.07)	[−0.12, 0.17]	0.702			
Multimorbidity (burden) × Education level (low)	−**0.25 (0.12)**	[−0.49, −0.01]	0.045			
Multimorbidity (burden) × Education level (medium)	−0.10 (0.11)	[−0.31, 0.11]	0.369			
Multimorbidity (burden) × Financial hardship (no)	**0.30 (0.09)**	[0.12, 0.47]	< 0.001			
Multimorbidity (burden) × Language (French)	−0.14 (0.08)	[−0.30, 0.03]	0.106			
Multimorbidity (burden) × Language (Italian)	−0.10 (0.17)	[−0.43, 0.24]	0.568			
Multimorbidity (count: yes) × Age				**0.69 (0.18)**	[0.34, 1.04]	< 0.001
Multimorbidity (count: yes) × Age^2^				−**0.00 (0.00)**	[−0.01, −0.00]	< 0.001
Multimorbidity (count: yes) × Sex (female)				0.35 (0.32)	[−0.27, 0.97]	0.270
Multimorbidity (count: yes) × Education level (low)				−**1.07 (0.52)**	[−2.09, −0.06]	0.039
Multimorbidity (count: yes) × Education level (medium)				−0.23 (0.44)	[−1.09, 0.63]	0.603
Multimorbidity (count: yes) × Financial hardship (no)				**1.77 (0.41)**	[0.96, 2.58]	< 0.001
Multimorbidity (count: yes) × Language (French)				−0.35 (0.36)	[−1.06, 0.36]	0.331
Multimorbidity (count: yes) × Language (Italian)				−1.16 (0.79)	[−2.70, 0.39]	0.142
**Random effects (variance components)**						
Random intercept (mergeid)	SD = 5.52; Var = 30.48			SD = 6.11; Var = 37.31		
Residual	SD = 7.08; Var = 50.20			SD = 7.28; Var = 53.03		

## Data Availability

The data used in this study are from the Survey of Health, Ageing and Retirement in Europe (SHARE). SHARE data are available free of charge for scientific use to registered users through the SHARE Research Data Center. Access is granted on an individual basis following registration and acceptance of the SHARE Conditions of Use. Information on data access and registration is available at: https://shareeric.eu/data/data-access. The SHARE data used in this study cannot be redistributed by the authors in accordance with the SHARE Conditions of Use. Statistical code is available from the corresponding author upon reasonable request.

## Abbreviations

ADL: Activities of daily living
ATHLOS: Ageing Trajectories of Health:Longitudinal Opportunities and Synergies
EURO-D: European Depression Scale
GBD: Global Burden of Disease
ICF: International Classification of Functioning, Disability and Health
IADL: Instrumental activities of daily living
IRT: Item response theory
ISCED: International Standard Classification of Education
LTC: Long-term care
NCDs: Noncommunicable diseases
SHARE: Survey of Health, Ageing and Retirement in Europe
STROBE: Strengthening the Reporting of Observational Studies in Epidemiology
UN: United Nations
WHO: World Health Organization.

## References

[1] Pahud O, Pellegrini S, Dorn M, Dutoit L, Zufferey J. Bedarf an Alters- und Langzeitpflege in der Schweiz. Aktualisierung der Bedarfsprognosen bis 2040 mit Blick bis 2050 (Obsan Bericht 09/2025). Neuchâtel: Schweizerisches Gesundheitsobservatorium; 2025.

[2] Chastonay P, Weber D, Mattig T. The health of older people in Switzerland. J Public Health Int. 2018;1(2):5–8. 10.14302/issn.2641-4538.jphi-18-2426.

[3] Chen QF, Ni C, Jiang Y, Chen L, Liao H, Gao J, Global burden of disease and its risk factors for adults aged 70 and older across 204 countries and territories: a comprehensive analysis of the Global Burden of Disease Study 2021. BMC Geriatr. 2025;25(1):462. 10.1186/s12877-025-06095-1. PMC12220231 40604507

[4] World Health Organization. Decade of healthy ageing: baseline report [Internet]. Geneva: World Health Organization. 2020. Available from: https://apps.who.int/iris/handle/10665/338677

[5] World Health Organization. Integrated care for older people (ICOPE): guidance for person-centred assessment and pathways in primary care. 2nd ed. Geneva: World Health Organization; 2024.

[6] Cesari M, Araujo de Carvalho I, Amuthavalli Thiyagarajan J, Cooper C, Martin FC, Reginster JY, Evidence for the Domains Supporting the Construct of Intrinsic Capacity. J Gerontol Biol Sci Med Sci. 2018;73(12):1653–60. 10.1093/gerona/gly011. 29408961

[7] Beard JR, Jotheeswaran AT, Cesari M, Araujo De Carvalho I. The structure and predictive value of intrinsic capacity in a longitudinal study of ageing. BMJ Open. 2019;9(11):e026119. 10.1136/bmjopen-2018-026119. PMC6830681 31678933

[8] Beard JR, Si Y, Liu Z, Chenoweth L, Hanewald K. Intrinsic Capacity: Validation of a New WHO Concept for Healthy Aging in a Longitudinal Chinese Study. J Gerontol Biol Sci Med Sci. 2022;77(1):94–100. 10.1093/gerona/glab226. 34343305

[9] Beard JR, Hanewald K, Si Y, Amuthavalli Thiyagarajan J, Moreno-Agostino D. Cohort trends in intrinsic capacity in England and China. Nat Aging. 2024;5(1):87–98. 10.1038/s43587-024-00741-w. PMC11754101 39702725

[10] Tan F, Wei X, Zhang J, Zhao Y, Tong X, Michel JP, The assessment and detection rate of intrinsic capacity deficits among older adults: a systematic review and meta-analysis. BMC Geriatr. 2024;24(1):485. 10.1186/s12877-024-05088-w. PMC11149255 38831281

[11] Chen M, Hanewald K, Si Y, Gu Y, Beard JR. Intrinsic Capacity Across 15 Countries in the Survey of Health, Aging, and Retirement in Europe. JAMA Netw Open. 2025;8(5):e259792. 10.1001/jamanetworkopen.2025.9792. PMC12070240 40354051

[12] Nishio M, Ito Y, Kondo N, Amuthavalli Thiyagarajan J. Towards a global scale for functional ability: what gets measured and gets done—but are we measuring the right thing? Age Ageing. 2025;54(11):afaf323. 10.1093/ageing/afaf323. PMC12596263 41206103

[13] World Health Organization. World report on ageing and health. Geneva: World Health Organization; 2015.

[14] World Health Organization. International classification of functioning, disability and health : ICF [Internet]. Geneva: World Health Organization. 2001. Available from: https://apps.who.int/iris/handle/10665/42407

[15] Hajek A, König HH. Longitudinal Predictors of Functional Impairment in Older Adults in Europe – Evidence from the Survey of Health, Ageing and Retirement in Europe. PLoS ONE. 2016;11(1):e0146967. 10.1371/journal.pone.0146967. PMC4718586 26784698

[16] Fors S, Illinca S, Jull J, Kadi S, Phillips P, Rodrigues S. Cohort-specific disability trajectories among older women and men in Europe 2004–2017. Eur J Ageing. 2022;19(4):1111–9. 10.1007/s10433-022-00684-4. PMC9729672 36506653

[17] Sanchez-Niubo A, Forero CG, Wu YT, Giné-Vázquez I, Prina M, De La Fuente J, Development of a common scale for measuring healthy ageing across the world: results from the ATHLOS consortium. Int J Epidemiol. 2021;50(3):880–92. 10.1093/ije/dyaa236. PMC8271194 33274372

[18] Nguyen H, Moreno-Agostino D, Chua KC, Vitoratou S, Prina AM. Trajectories of healthy ageing among older adults with multimorbidity: A growth mixture model using harmonised data from eight ATHLOS cohorts. Zhan Y, editor. PLOS ONE. 2021;16(4):e0248844. 10.1371/journal.pone.0248844 PMC8023455 33822803

[19] Fellinghauer C, Hodel J, Moreira B, Kim J, Sabariego C. Development of a functioning metric for the ageing population using data from the survey of health, ageing and retirement in Europe (SHARE). Christensen KB, editor. PLOS One. 2025;20(4):e0320068. 10.1371/journal.pone.0320068 PMC12021154 40273079

[20] Hodel J, Rothacher Y, Moreira B, Fellinghauer C, Pacheco Barzallo D, Weisstanner D, Disability severity transitions in the community-dwelling Swiss ageing population: secondary analysis of the Swiss version of the Survey of Health, Ageing and Retirement in Europe (SHARE) using multistate models. BMJ Open. 2026;16(2):e104871. 10.1136/bmjopen-2025-104871. PMC12911770 41689217

[21] Nicholson K, Liu W, Fitzpatrick D, Hardacre KA, Roberts S, Salerno J, Prevalence of multimorbidity and polypharmacy among adults and older adults: a systematic review. Lancet Healthy Longev. 2024;5(4):e287–96. 10.1016/S2666-7568(24)00007-2. 38452787

[22] Ren Z, Xu Y, Sun J, Han Y, An L, Liu J. Chronic diseases and multimorbidity patterns, their recent onset, and risk of new-onset Parkinson’s disease and related functional degeneration in older adults: a prospective cohort study. eClinicalMedicine. 2023;65:102265. 10.1016/j.eclinm.2023.102265. PMC10579290 37855021

[23] Bayes-Marin I, Sanchez-Niubo A, Egea-Cortés L, Nguyen H, Prina M, Fernández D, Multimorbidity patterns in low-middle and high income regions: a multiregion latent class analysis using ATHLOS harmonised cohorts. BMJ Open. 2020;10(7):e034441. 10.1136/bmjopen-2019-034441. PMC7371222 32690500

[24] Nguyen H, Manolova G, Daskalopoulou C, Vitoratou S, Prince M, Prina AM. Prevalence of multimorbidity in community settings: A systematic review and meta-analysis of observational studies. J Comorbidity. 2019;9:2235042X19870934. 10.1177/2235042X19870934. PMC6710708 31489279

[25] Excoffier S, Herzig L, N’Goran AA, Déruaz-Luyet A, Haller DM. Prevalence of multimorbidity in general practice: a cross-sectional study within the Swiss Sentinel Surveillance System (Sentinella). BMJ Open. 2018;8(3):e019616. 10.1136/bmjopen-2017-019616. PMC5855161 29511015

[26] Moreira B, Hodel J, Selb M, Kim J, Fellinghauer C, Bickenbach J, Overview of Available Functioning Data in Switzerland: Supporting the Use of Functioning as a Health Indicator Alongside Mortality and Morbidity. Int J Public Health. 2024;69:1607366. 10.3389/ijph.2024.1607366. PMC11349544 39206139

[27] SHARE-ERIC. Survey of Health, Ageing and Retirement in Europe (SHARE) Wave 1. Release version: 9.0.0 [dataset]. SHARE-ERIC; 2024. 10.6103/SHARE.W1.900

[28] SHARE-ERIC. Survey of Health, Ageing and Retirement in Europe (SHARE) Wave 2. Release version: 9.0.0 [dataset]. SHARE-ERIC; 2024. 10.6103/SHARE.W2.900

[29] SHARE-ERIC. Survey of Health, Ageing and Retirement in Europe (SHARE) Wave 4. Release version: 9.0.0 [dataset]. SHARE-ERIC; 2024. 10.6103/SHARE.W4.900

[30] SHARE-ERIC. Survey of Health, Ageing and Retirement in Europe (SHARE) Wave 5. Release version: 9.0.0 [dataset]. SHARE-ERIC; 2024. 10.6103/SHARE.W5.900

[31] SHARE-ERIC. Survey of Health, Ageing and Retirement in Europe (SHARE) Wave 6. Release version: 9.0.0 [dataset]. SHARE-ERIC; 2024. 10.6103/SHARE.W6.900

[32] SHARE-ERIC. Survey of Health, Ageing and Retirement in Europe (SHARE) Wave 7. Release version: 9.0.0 [dataset]. SHARE-ERIC; 2024. 10.6103/SHARE.W7.900

[33] SHARE-ERIC. Survey of Health, Ageing and Retirement in Europe (SHARE) Wave 8. Release version: 9.0.0 [dataset]. SHARE-ERIC; 2024. 10.6103/SHARE.W8.900

[34] SHARE-ERIC. Survey of Health, Ageing and Retirement in Europe (SHARE) Wave 9. Release version: 9.0.0 [dataset]. SHARE-ERIC; 2024. 10.6103/SHARE.W9.900

[35] Bergmann M, Kneip T, Le M, De Luca G, Quezada M. Household and Individual Participation, Attrition, and Sample Development in the Survey of Health, Ageing and Retirement in Europe (SHARE) Waves 1–9. Based on Release 9.0.0. SHARE Working Paper Series 104–2025. Berlin: SHARE-ERIC; 2025. 10.6103/1042025

[36] Börsch-Supan A, Brandt M, Hunkler C, Kneip T, Korbmacher J, Malter F, Data Resource Profile: The Survey of Health, Ageing and Retirement in Europe (SHARE). Int J Epidemiol. 2013;42(4):992–1001. 10.1093/ije/dyt088. PMC3780997 23778574

[37] Masters GN. A Rasch Model for Partial Credit Scoring. Psychometrika. 1982;47(2):149–74. 10.1007/BF02296272.

[38] Prince MJ, Reischies F, Beekman ATF, Fuhrer R, Jonker C, Kivela SL, Development of the EURO–D scale – a European Union initiative to compare symptoms of depression in 14 European centres. Br J Psychiatry. 1999;174(4):330–8. 10.1192/bjp.174.4.330. 10533552

[39] Global Burden of Disease Collaborative Network. Global Burden of Disease Study 2021. (GBD 2021) Disability Weights. Seattle, United States: Institute for Health Metrics and Evaluation (IHME), 2024. Available from: https://www.healthdata.org

[40] Hosseinpoor AR, Stewart Williams J, Jann B, Kowal P, Officer A, Posarac A, Social determinants of sex differences in disability among older adults: a multi-country decomposition analysis using the World Health Survey. Int J Equity Health. 2012;11(1):52. 10.1186/1475-9276-11-52. PMC3463479 22958712

[41] Fabbri E, Zoli M, Gonzalez-Freire M, Salive ME, Studenski SA, Ferrucci L. Aging and Multimorbidity: New Tasks, Priorities, and Frontiers for Integrated Gerontological and Clinical Research. J Am Med Dir Assoc. 2015;16(8):640–7. 10.1016/j.jamda.2015.03.013. PMC5125299 25958334

[42] Beard JR, Officer A, De Carvalho IA, Sadana R, Pot AM, Michel JP, The World report on ageing and health: a policy framework for healthy ageing. Lancet. 2016;387(10033):2145–54. 10.1016/S0140-6736(15)00516-4. PMC4848186 26520231

[43] Darin-Mattsson A, Fors S, Kåreholt I. Different indicators of socioeconomic status and their relative importance as determinants of health in old age. Int J Equity Health. 2017;16(1):173. 10.1186/s12939-017-0670-3. PMC5615765 28950875

[44] Stekhoven DJ, Bühlmann P. MissForest—non-parametric missing value imputation for mixed-type data. Bioinformatics. 2012;28(1):112–8. 10.1093/bioinformatics/btr597. 22039212

[45] Lusa L, Proust-Lima C, Schmidt CO, Lee KJ, Le Cessie S, Baillie M, Initial data analysis for longitudinal studies to build a solid foundation for reproducible analysis. PLoS ONE. 2024;19(5):e0295726. 10.1371/journal.pone.0295726. PMC11135704 38809844

[46] Pinheiro J, Bates D, R Core Team. nlme: Linear and Nonlinear Mixed Effects Models. R package version 3.1–168 [Internet]. 2025 [cited 2026 Feb 16]. Available from: https://CRAN.R-project.org/package=nlme.10.32614/CRAN.package.nlme

[47] Von Elm E, Altman DG, Egger M, Pocock SJ, Gøtzsche PC, Vandenbroucke JP, The Strengthening the Reporting of Observational Studies in Epidemiology (STROBE) Statement: Guidelines for Reporting Observational Studies. PLoS Med. 2007;4(10):e296. 10.1371/journal.pmed.0040296. PMC2020495 17941714

[48] World Health Organization. Framework to implement a life course approach in practice. Geneva: World Health Organization; 2025.

[49] Chang AY, Skirbekk VF, Tyrovolas S, Kassebaum NJ, Dieleman JL. Measuring population ageing: an analysis of the Global Burden of Disease Study 2017. Lancet Public Health. 2019;4(3):e159–67. 10.1016/S2468-2667(19)30019-2. PMC6472541 30851869

[50] World Health Organization. The Global Health Observatory. Geneva: World Health Organization; 2015.

[51] Calderón-Larrañaga A, Vetrano DL, Ferrucci L, Mercer SW, Marengoni A, Onder G, Multimorbidity and functional impairment–bidirectional interplay, synergistic effects and common pathways. J Intern Med. 2019;285(3):255–71. 10.1111/joim.12843. PMC6446236 30357990

[52] Bundesamt für Gesundheit (BAG). NCD-Strategie: Auftrag, Ziele, Massnahmen und Steuerung [Internet]. Bern: Bundesamt für Gesundheit; [cited 2026 Aug 13]. Available from: https://www.bag.admin.ch/bag/de/home/strategie-und-politik/nationale-gesundheitsstrategien/strategie-nichtuebertragbare-krankheiten/auftrag.html

[53] World Health Organization. Rehabilitation: a key strategy in addressing noncommunicable diseases. Policy brief. 2025 Feb. Available from: https://cdn.who.int/media/docs/default-source/documents/health-topics/rehabilitation/policy-brief-rehabilitation-a-key-strategy-in-addressing-ncds.pdf

[54] Makovski TT, Cuschieri S, Valderas JM, Guldner L, Nagyova I, Van Den Akker M, Preventing Multimorbidity: Moving Beyond the Single Disease Lens. Int J Public Health. 2025;70:1608880. 10.3389/ijph.2025.1608880. PMC12436202 40963513

[55] Wagg E, Blyth FM, Cumming RG, Khalatbari-Soltani S. Socioeconomic position and healthy ageing: A systematic review of cross-sectional and longitudinal studies. Ageing Res Rev. 2021;69:101365. 10.1016/j.arr.2021.101365. 34004378

[56] Wu YT, Daskalopoulou C, Muniz Terrera G, Sanchez Niubo A, Rodríguez-Artalejo F, Ayuso-Mateos JL, Education and wealth inequalities in healthy ageing in eight harmonised cohorts in the ATHLOS consortium: a population-based study. Lancet Public Health. 2020;5(7):e386–94. 10.1016/S2468-2667(20)30077-3. PMC7739372 32619540

[57] Boes S, Weisstanner D, Durvy B. Health Systems in Action (HSiA) Insights – Switzerland, 2024. Copenhagen: European Observatory on Health Systems and Policies, WHO Regional Office for Europe; 2025.

[58] Nguyen H, Moreno-Agostino D, Chua KC, Vitoratou S, Prina AM. Trajectories of healthy ageing among older adults with multimorbidity: A growth mixture model using harmonised data from eight ATHLOS cohorts. Zhan Y, editor. PLOS ONE. 2021;16(4):e0248844. 10.1371/journal.pone.0248844 PMC8023455 33822803

[59] Huisman M, Kunst AE, Mackenbach JP. Socioeconomic inequalities in morbidity among the elderly; a European overview. Soc Sci Med. 2003;57(5):861–73. 10.1016/S0277-9536(02)00454-9. 12850111

[60] Tooth L, Hockey R, Byles J, Dobson A. Weighted multimorbidity indexes predicted mortality, health service use, and health-related quality of life in older women. J Clin Epidemiol. 2008;61(2):151–9. 10.1016/j.jclinepi.2007.05.015. 18177788

[61] Bickenbach J, Rubinelli S, Baffone C, Stucki G. The human functioning revolution: implications for health systems and sciences. Front Sci. 2023;1:1118512. 10.3389/fsci.2023.1118512.

[62] Bundesamt für Statistik (BFS). Bevölkerung nach Migrationsstatus [Internet]. Neuchâtel: Bundesamt für Statistik; [cited 2026 Aug 13]. Available from: https://www.bfs.admin.ch/bfs/en/home/statistics/population/migration-integration/by-migration-status.html

